# Detecting the causality influence of individual meteorological factors on local PM_2.5_ concentration in the Jing-Jin-Ji region

**DOI:** 10.1038/srep40735

**Published:** 2017-01-27

**Authors:** Ziyue Chen, Jun Cai, Bingbo Gao, Bing Xu, Shuang Dai, Bin He, Xiaoming Xie

**Affiliations:** 1 College of Global Change and Earth System Science, Beijing Normal University, 19 Xinjiekouwai Street, Haidian, Beijing 100875, P. R. China; 2 Joint Center for Global Change Studies, Beijing 100875, China; 3 Department of Earth System Science, Tsinghua University, Beijing 100084, China; 4 National Engineering Research Center for Information Technology in Agriculture, 11 Shuguang Huayuan Middle Road, Beijing 100097, China

## Abstract

Due to complicated interactions in the atmospheric environment, quantifying the influence of individual meteorological factors on local PM_2.5_ concentration remains challenging. The Beijing-Tianjin-Hebei (short for Jing-Jin-Ji) region is infamous for its serious air pollution. To improve regional air quality, characteristics and meteorological driving forces for PM_2.5_ concentration should be better understood. This research examined seasonal variations of PM_2.5_ concentration within the Jing-Jin-Ji region and extracted meteorological factors strongly correlated with local PM_2.5_ concentration. Following this, a convergent cross mapping (CCM) method was employed to quantify the causality influence of individual meteorological factors on PM_2.5_ concentration. The results proved that the CCM method was more likely to detect mirage correlations and reveal quantitative influences of individual meteorological factors on PM_2.5_ concentration. For the Jing-Jin-Ji region, the higher PM_2.5_ concentration, the stronger influences meteorological factors exert on PM_2.5_ concentration. Furthermore, this research suggests that individual meteorological factors can influence local PM_2.5_ concentration indirectly by interacting with other meteorological factors. Due to the significant influence of local meteorology on PM_2.5_ concentration, more emphasis should be given on employing meteorological means for improving local air quality.

Recent studies^1^,^2^,^3^,^4^,^5^ proved that airborne pollutants, PM_2.5_ in particular, were closely related to all-cause and specific-cause mortality. In this case, increasing efforts have been made on regular monitoring of air quality. Furthermore, general public and local governments in China are placing growing emphasis on a better understanding of airborne pollutants. Since the outbreak of frequent smog events in China since 2012, massive studies have been conducted recently to analyze sources^6^,^7^,^8^,^9^, characteristics^7^,^10^,^11^,^12^,^13^,^14^,^15^,^16^ and seasonal variations^17^,^18^,^19^,^20^,^21^,^22^,^23^,^24^ of PM_2.5_ in China. To map spatial variations of PM_2.5_ concentration across large areas, some researchers^25^,^26^ employed different remote sensing sources and spatial data analysis methods.

Among these studies, a large body of research has been conducted to examine the correlations between meteorological factors and airborne pollutants. Blanchard *et al*.^27^ indicated a near-linear correlation between ozone concentration and temperature and relative humidity, as well as some non-linear correlations between ozone and other meteorological factors. Juneng *et al*.^28^ suggested that local meteorological factors, especially local temperature, humidity and wind speed, dominated the fluctuation of PM_10_ over the Klang Valley during the summer monsoon. Pearce *et al*.^29^ quantified the influence of local meteorology on air quality and the result indicated that the meteorology at the local-scale, was a relatively strong driver for the air quality in Melbourne. This research found that local temperature led to strongest responses from different airborne pollutants, whilst other meteorological factors mainly affected one or more pollutant types. Galindo *et al*.^30^ found that fractions of three different sizes were negatively correlated with winter wind speed, whilst the temperature and solar radiation had strong influences on coarse fractions. El-Metwally and Alfaro^31^ pointed out that the wind speed was related to both the dilution and the composition of airborne pollutants. Grundstrom *et al*.^32^ proved that low wind speeds and positive vertical temperature gradients were high risk factors for elevated NOx and particle number concentrations (PNC). Zhang *et al*.^14^ assessed the relationship between meteorological factors and critical air pollutants in Beijing, Shanghai and Guangzhou, and confirmed that the role of meteorological factors in airborne pollutant formation varied significantly across different seasons and geological locations.

However, the analysis of the sensitivity of airborne pollutants to individual meteorological parameters remains particularly difficult^29^, as different meteorological parameters are inherently linked and may affect airborne pollutants through both direct and indirect mechanisms. In this case, Pearce *et al*.^29^ suggested that multiple models and methods should be comprehensively considered to quantify the role of meteorological factors in affecting local air pollution.

The Beijing-Tianjin-Hebei (or referred to as Jing-Jin-Ji) region, located in the north of North China Plain, is one of the most influential regions in China. The Jing-Jin-Ji region consists of a series of cities, including Beijing, Tianjin, Baoding, Langfang, Tangshan, Zhangjiakou, Chengde, Qinhuangdao, Cangzhou, Hengshui, Xingtai, Handan (Due to lack of consistent meteorological data, this city is not included in this research) and Shijiazhuang. Geographical locations of these cities are demonstrated in Fig. 1.

Chan and Yao^33^ pointed out that the Jing-Jin-Ji region experienced most serious airborne pollution in China, which was further proved by frequent regional smog events since 2012. To better forecast and enhance local air quality within the Jing-Jin-Ji region, it is necessary to gain a better understanding of the characteristics of PM_2.5_ concentration and the meteorological influences on PM_2.5_ concentration. To this end, characteristics and seasonal variations of PM_2.5_ concentration in this region are analyzed. Next, correlations between a set of individual meteorological factors and PM_2.5_ concentration are examined, and those meteorological factors strongly correlated with PM_2.5_ concentration are extracted for each city. Following the correlation analysis, a convergent cross mapping (CCM) method is employed to quantify the causality influence of these extracted individual meteorological factors on PM_2.5_ concentration. Hence, the performance of correlation and causality analysis in complicated atmospheric environment can be comprehensively compared. Based on these analysis, this research aims to not only quantify the meteorological influences on PM_2.5_ concentration within the Jing-Jin-Ji region, but also provides useful reference for mitigating air pollution in other areas.

## Results

### Characteristics and variations of PM_2.5_ concentration within the Jing-Jin-Ji region

**Figure 2:**
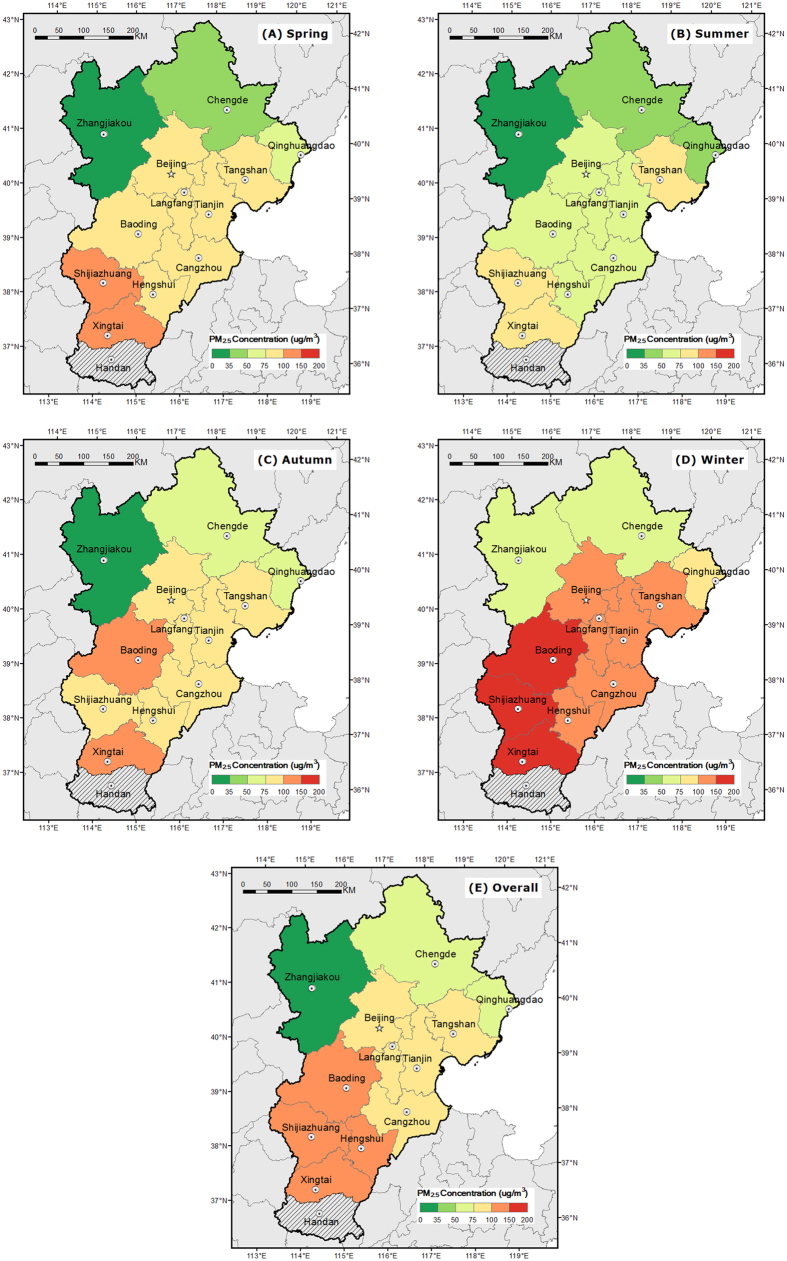
Seasonal and overall mean daily PM_2.5_ concentration for different cities in the Jing-Jin-Ji region. The maps were drawn by the software of ArcGIS version 10.2, http://www.esri.com/software/arcgis/arcgis-for-desktop.

For the study period between Jan 8^th^, 2014 and Dec 31^st^, 2014, daily PM_2.5_ concentration for main cities in the Jing-Jin-Ji region was analyzed respectively. Previous studies^14^,^21^,^34^ proved that air quality in China was of notable seasonal variations. In this study, PM_2.5_ concentration is also analyzed for each season respectively. In the Jing-Jin-Ji region, central heating is provided for cities during Nov 15^th^ to March 15^th^. Thus this period is commonly categorized as winter for this region. According to the characteristics of high temperature, the period from June 1^st^ to August 31^st^ is defined as the summer. Accordingly, spring is defined as the period from March 16^th^ to May 31^st^ whilst autumn is defined as the period between September 1^st^ and Nov 14^th^. The criteria for categorizing four seasons are consistent with a common phenomenon in Beijing, which is described by old sayings as “The spring and autumn in Beijing hardly last long”. General characteristics of PM_2.5_ concentration for different cities are demonstrated as Table 1 and Fig. 2.

As shown in Table 1 and Fig. 2, it is noted that general PM_2.5_ concentration in the Jing-Jin-Ji region is much higher than Global Guidelines set by the World Health Organization (WHO) (24-hour mean: 25 μg/m^3^). As concluded by previous studies^21^,^22^, PM_2.5_ concentration for Beijing is the highest in winter. This phenomenon also applies to other cities in the Jing-Jin-Ji region. The notably deteriorated air quality in winter may mainly attribute to the fact that central heating by burning coal materials, is supplied widely for the Jing-Jin-Ji region and thus leads to extra emission of airborne pollutants. According to PM_2.5_ concentration, the Jing-Jin-Ji region can be divided into three sub-regions; slightly polluted region: Zhangjiakou, Chengde, Qinghuangdao; moderately polluted region: Beijing, Langfang, Tangshan, Tianjin, Cangzhou; heavily polluted region: Baoding, Hengshui, Xingtai, Shijiazhuang.

### Meteorological factors correlated with PM_2.5_ concentration

Based on a case study in Beijing, Shanghai and Guangzhou, Zhang *et al*.^14^ suggested that relative humidity, temperature, wind speed and wind directions were main meteorological factors correlated with the concentration of airborne pollutants. In addition, some other scholars^29^,^30^,^31^,^32^,^33^,^34^,^35^ pointed out that radiation, evaporation, precipitation and air pressure also influenced PM_2.5_ concentration. Therefore, to comprehensively understand meteorological driving forces for PM_2.5_ concentration in the Jing-Jin-Ji region, a set of factors was selected as follows: evaporation, temperature, wind, precipitation, radiation, humidity, and air pressure. To better analyze the role of these meteorological factors in affecting local PM_2.5_ concentration, these factors are further categorized into sub-factors: evaporation (small evaporation and large evaporation, short for smallEVP and largeEVP), temperature (daily max temperature, mean temperature and min temperature, short for maxTEM, meanTEM and minTEM), precipitation (total precipitation from 8am–20pm and total precipitation from 20pm–8am, short for PRE8-20, PRE20-8), air pressure (daily max pressure, mean pressure and min pressure, short for maxPRS, meanPRS and minPRS), humidity (daily mean and min relative humidity, short for meanRHU and minRHU), solar radiation (daily sunshine duration, short for SSD) and wind (daily mean wind speed, max wind speed, extreme wind speed and max wind direction, short for meanWIN, maxWIN, extWIN and dir_maxWIN). As there are one or more observation stations for each city, the daily value for meteorological factors for each city was acquired by averaging the value from all available observation stations.

Through correlation analysis, meteorological factors strongly correlated with PM_2.5_ concentration were extracted for each city (Table 2). According to Table 2, meteorological factors strongly correlated with PM_2.5_ concentration were of notable characteristics in different seasons. PM_2.5_ concentration was the highest in winter and there were more influential meteorological factors on PM_2.5_ concentration in winter. Additionally, there was no meteorological factor strongly correlated with PM_2.5_ concentration for all cities or all seasons. In this case, it is more meaningful to analyze correlations between meteorological factors and PM_2.5_ concentration on a seasonal basis rather than an annual basis.

Due to complicated interactions between different meteorological factors in the atmospheric environment, correlation analysis may extract mirage correlations. Additionally, the value of correlation coefficients cannot directly reflect the quantitative influence of individual meteorological factors on PM_2.5_ concentration. However, correlated meteorological factors provide important reference for the following causality analysis. Although the correlation between two variables does not guarantee their causality, two coupled variables (except for some weak coupling) are usually correlated. Therefore, meteorological factors correlated with PM_2.5_ concentration are further selected for the causality analysis.

### The causality influence of individual meteorological factors on local PM_2.5_ concentration

By analyzing two time-series variables using the CCM method, researchers can understand their coupling according to an output convergent map. If the interaction between two variables is featured using generally convergent curves with increasing time series length, then the causality is detected. On the other hand, if the interaction between the two variables is featured as curves without any general trend, then no causality exists between the two variables. The value of predictive skills (denoted by *ρ* value), ranging from 0 to 1, presents the strength of influences from one variable on another variable. The CCM method is highly automatic and detailed parameter setting for this model is explained in the method section.

**Figure 3:**
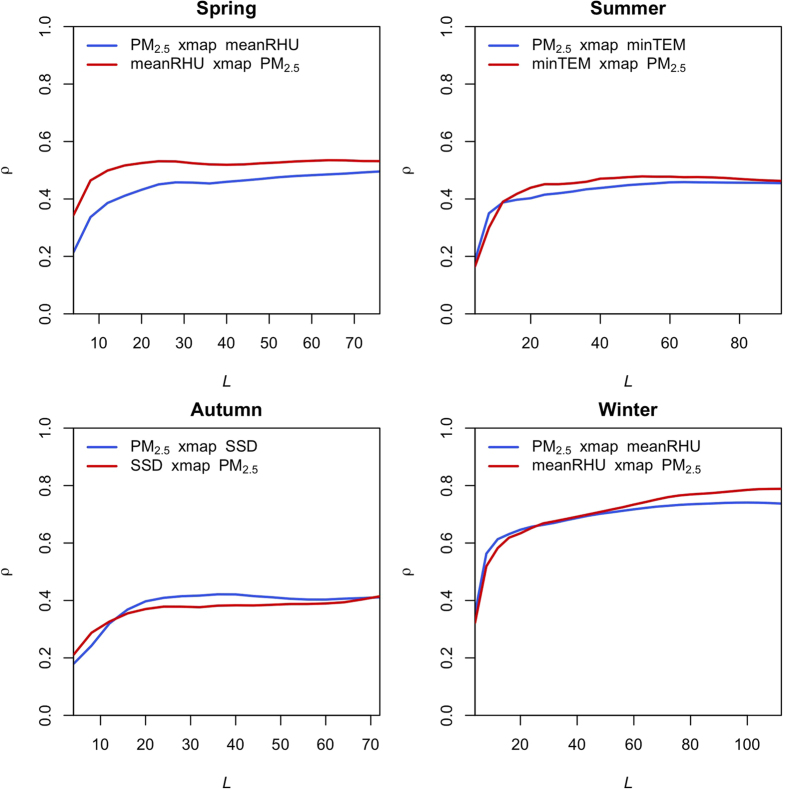
Some illustrative CCM results to demonstrate the causality between meteorological factors and PM_2.5_ concentration in Beijing. *ρ*: predictive skills. *L*: the length of time series. A xmap B stands for convergent cross mapping B from A, in other words, the causality influence of variable B on A. For instance, PM_2.5_ xmap meanRHU stands for the causality influence of meanRHU on PM_2.5_ concentration. *ρ* indicates the predictive skills of using meanRHU to retrieve PM_2.5_ concentration.

The quantitative coupling between PM_2.5_ concentration and individual meteorological factors is explained using convergent cross maps. Thus, there should be a convergent cross map for each variable in Table 2. It is not feasible to present more than 100 convergent maps here to explain the causality between PM_2.5_ concentration and each meteorological factor respectively. Hence several convergent cross maps (Fig. 3) are displayed to demonstrate how CCM method works. For the rest causalities, Table 2 is presented to explain the quantitative influence of each meteorological factor on PM_2.5_ concentration (*ρ* value). It is worth mentioning that *ρ* value can be extracted through the CCM tool directly, instead of the visual interpretation of the convergent cross map. If *ρ* is convergent to a certain value (in other words, Δ*ρ* is approaching to 0) with increasing time series, then the causality is detected and the ultimate *ρ* value for the coupling is set as the convergent constant. The *ρ* extraction approach based on computation allows the application of the CCM method to a national or global scale, where a diversity of interactions between variables should be examined.

As Fig. 3 demonstrates, the coupling between meteorological factors and PM_2.5_ concentration can be bidirectional. On one hand, some meteorological factors have important influences on PM_2.5_ concentration. On the other hand, PM_2.5_ concentration has significant feedback effects on these meteorological factors. Therefore, the meteorological factor can continuously influence local PM_2.5_ concentration through even more complicated processes. For instance, local meanRHU has a strong influence (*ρ* = 0.738) on Beijing PM_2.5_ concentration in winter whilst local PM_2.5_ concentration has a strong feedback effect (*ρ* = 0.786) on meanRHU. Unlike GC analysis, the CCM method does not indicate the positive or negative causality between two variables directly. However, taking the correlation analysis into account, it is known that meanRHU has a positive influence on PM_2.5_ concentration whilst PM_2.5_ concentration has a positive feedback on meanRHU. In this case, high meanRHU in Beijing is more likely to cause high PM_2.5_ concentration, which results in even higher meanRHU. In turn, higher meanRHU can further increase local PM_2.5_ concentration. By analogy, the process how other meteorological factors influence local PM_2.5_ concentration can be understood as well.

Table 2 suggests that the causality influence of individual meteorological factors on PM_2.5_ concentration is better revealed using the CCM method than the correlation analysis. By comparing the correlation coefficient and *ρ* value in Table 2, one can see that some correlations between meteorological factors and PM_2.5_ concentration may result from mirage correlations (e.g. the correlation between meanRHU and PM_2.5_ concentration in Hengshui in summer). Secondly, CCM analysis reveals weak or moderate coupling (e.g. the interactions between SSD and PM_2.5_ concentration in Cangzhou in summer) whilst correlation analysis cannot. Additionally, due to interactions between different meteorological factors, the value of correlation coefficients cannot interpret the quantitative influence of individual meteorological factors on PM_2.5_ concentration. Instead, the *ρ* value from CCM method is designed to understand the coupling between two variables by excluding influences from other factors. Through comparison, the value of the correlation coefficient for some meteorological factors is notably different from the *ρ* value for these meteorological factors. A large correlation coefficient for one meteorological factor may correspond to a much smaller *ρ* value from the CCM analysis (e.g. the correlation and causality between smallEVP and PM_2.5_ concentration in Beijing in winter).

Although some limitations exist, correlation analysis provides valuable reference for understanding the relationship between PM_2.5_ concentration and meteorological factors. Firstly, the CCM method cannot directly indicate positive or negative causality between two variables. In this case, the correlation coefficient (with “+” or “−”) provides researchers with a possible way to understand the causality direction. Secondly, even if the correlation coefficient is not an indicator of quantitative causality, it can be employed as a qualitative indicator for understanding the interactions between PM_2.5_ concentration and meteorological factors. Based on Table 2, it is noted that except for very few mirage correlations, meteorological factors strongly correlated with PM_2.5_ concentration, also have a causality influence on PM_2.5_ concentration. If the research objective is to simply extract meteorological factors that influence PM_2.5_ concentration and the analysis of quantitative influences is not required, then the correlation analysis can be an alternative approach (with a small possibility of mirage correlations) for analyzing the qualitative relationship between PM_2.5_ concentration and individual meteorological factors.

**Figure 4:**
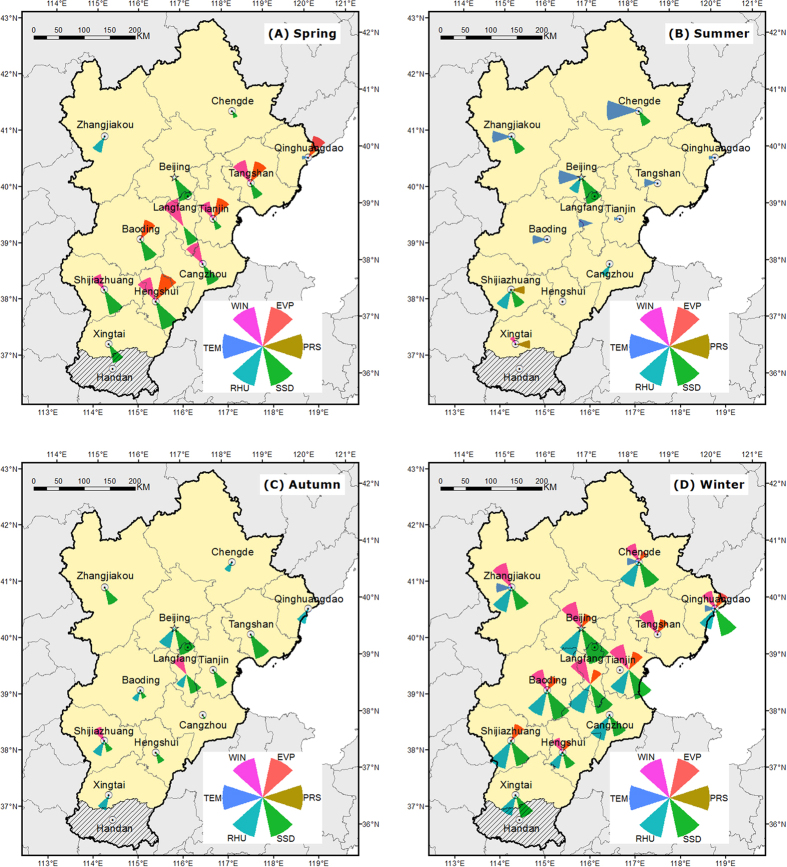
Seasonal influences of individual meteorological factors on PM_2.5_ concentration for different cities within Jing-Jin-Ji region. The size of the wind rose petal in the legend is decided by the maximum *ρ* value, 1.0. And the size of the wind rose petal on the map represents the actual *ρ* value of the specific meteorological influences on local PM_2.5_ concentration. The maps were drawn by the software of ArcGIS version 10.2, http://www.esri.com/software/arcgis/arcgis-for-desktop.

To properly demonstrate the influence of different meteorological factors on local PM_2.5_ concentration, a wind rose was produced for each city through R programming. Firstly, a histogram featuring *ρ* value of each meteorological factor was produced. Next, according to the maximum of *ρ* value of each meteorological factor, the range of y axis was decided. Finally, a wind rose was made by transforming the histogram into polar-formed graph. Thus, seasonal wind rose maps that feature the causality influence (*ρ* value) of individual meteorological factors on PM_2.5_ concentration in the Jing-Jin-Ji region are shown as Fig. 4.

Compared with Table 2, Fig. 4 presents seasonal influences of individual meteorological factors on local PM_2.5_ concentration using easily understandable maps. According to these wind rose maps, some notable characteristics can be found:

- PM_2.5_ concentration in winter is notably higher than that in other seasons. Accordingly, the number of meteorological factors that influence PM_2.5_ concentration in winter is more than that in other seasons. Furthermore, the quantitative influence (*ρ* value) of meteorological factors on PM_2.5_ concentration in winter is much stronger than that in other seasons. On the other hand, PM_2.5_ concentration in summer is the lowest and there are fewer meteorological factors that influence PM_2.5_ concentration than in other seasons. The meteorological influences on PM_2.5_ concentration in summer are also smaller than other seasons. This phenomenon is consistent with strong coupling between PM_2.5_ concentration and meteorological factors, as explained above. The higher PM_2.5_ concentration, the stronger influences it exerts on meteorological factors. In turn, corresponding meteorological factors can have a stronger feedback effect on PM_2.5_ concentration.
- There is no meteorological factor that consistently influences PM_2.5_ concentration across seasons. In summer, the PM_2.5_ concentration is the lowest and there are very limited meteorological factors that influence PM_2.5_ concentration notably. The meteorological factor, temperature (especially minTEM), which has little influence on PM_2.5_ concentration in other seasons, plays a dominant role in influencing PM_2.5_ concentration in summer. In winter, PM_2.5_ concentration is the highest and there are many meteorological factors that significantly influence PM_2.5_ concentration. It is difficult to extract one dominant influential meteorological factor for PM_2.5_ concentration, as Humidity, SSD and Wind work together to exert significant influences on PM_2.5_ concentration in winter.
- The correlation between some meteorological factors (temperature, wind and humidity) and air quality in big cities in China has been well discussed by previous studies^14^. However, the role of radiation is not considered fully. As shown in Fig. 4, SSD exerts notable influences on PM_2.5_ concentration in all seasons, especially in winter. As a result, more emphasis should be given on understanding the role of radiation in influencing local PM_2.5_ concentration.

## Discussion

Although the CCM method proved the causality between PM_2.5_ concentration and individual meteorological factors, it did not explain why these variables were interacted. To better understand meteorological influences on PM_2.5_ concentration and its feedback effects, we attempt to explain the mechanisms of some typical bidirectional coupling.

Wind, humidity and SSD are the most influential meteorological factors for PM_2.5_ concentration in winter. Herein, we take the three factors as example to briefly explain underlying interactions between meteorological factors and PM_2.5_ concentration.

### Negative bidirectional coupling between wind and PM_2.5_ concentration

On one hand, winds, especially strong winds blow airborne pollutants away and reduce PM_2.5_ concentration effectively. On the other hand, high PM_2.5_ concentration, especially a quickly rising PM2.5 concentration brings the atmospheric environment to a comparatively stable status, which prevents the form of winds and reduces the wind speed in smog-covered areas.

### Positive bidirectional coupling between humidity and PM_2.5_ concentration

higher humidity causes more vapors attached to the Particulate Matter (PM) and significantly increases the size and mass concentration of PM, namely the hygroscopic increase and accumulation of PM_2.5_^36^. On the other hand, the larger mass and higher concentration makes it difficult for PM_2.5_ to disperse and leads to a stable polluted atmospheric environment, which is not favorable for the vapor evaporation and further increases the environmental humidity.

### Negative bidirectional coupling between SSD and PM_2.5_ concentration

Previous studies^7^,^9^ have proved that organic carbon (OC) is an important component for PM_2.5,_ and atmospheric photolysis could occur on OC to reduce PM_2.5_ concentration. Therefore, longer SSD has a negative influence on PM_2.5_ concentration. On the other hand, SSD is a general indicator of cloudiness (https://en.wikipedia.org/wiki/Sunshine_duration). The more cloud, the less SSD is recorded by the ground observation station. By analogy, serious smog (thick black fog) caused by high PM_2.5_ concentration notably blocked radiation emitted to the ground and thus the PM_2.5_ concentration has a negative feedback effect on the SSD.

High PM_2.5_ concentration in the Jing-Jin-Ji region makes the improvement of air quality a top priority for central and local governments. Taking Beijing for instance, we explain why and how to employ meteorological means for improving air quality. A series of traffic and industrial restriction regulations has been proposed in recent years and the air quality in Beijing has been improved significantly. However, PM_2.5_ concentration in Beijing remains much higher than standard recommended by the WHO. In this case, as well as economic and administrative means, growing emphasis should be given on improving air quality through meteorological means. Meanwhile, some scholars suggested that meteorological factors were external driving forces whilst the exhaust of traffic and industry pollutants was the fundamental reason for high PM_2.5_ concentration. Therefore, adjusting meteorological factors was not the essential and most effective approach for mitigating local PM_2.5_ concentration.

Although these arguments all make sense, based on findings of our previous work^22^ and this research, enhancing air quality through meteorological means can be highly effective. Chen, Z. *et al*.^22^ found that air quality in Beijing experienced frequent sudden changes throughout a year. During Jan 8^th^, 2014 to Jan 7^th^, 2015, there were more than 180 days that experienced notable air quality change (air quality index difference, ΔAQI ≥ 50). Considering that the amount of traffic and industry induced exhaust is unlikely to change significantly on a daily basis, meteorological influences on daily PM_2.5_ concentration are crucial. This research further supports this hypothesis. The smog weather, resulting from high PM_2.5_ concentration, occurs most frequently in winter. Meanwhile, according to Table 2 and Fig. 4, the coupling between meteorological factors and PM_2.5_ concentration is the strongest in winter.

In addition to influence PM_2.5_ concentration directly, individual meteorological factors can indirectly influence PM_2.5_ concentration by interacting with other meteorological factors. Taking the wind factor for instance. in winter, three meteorological factors, humidity, wind and radiation (SSD) all strongly influence PM_2.5_ concentration in Beijing. As well as the direct influence (*ρ* > 0.5), the wind factor influences local PM_2.5_ concentration through some indirect mechanisms. Through correlation and causality analysis, quantitative interactions between wind and other factors in winter were summarized as follows:

- The correlation coeffienct between maxWIN and SSD was 0.508** and the quantitative influence of maxWIN on SSD (*ρ* value) was 0.362. So wind factor has a strong positive influence on SSD. (The mechanism for the positive influence of wind on SSD may not be evident, so a brief explanation is given here. As introduced above, SSD is the general indicator of cloudiness. The fewer clouds, the higher SSD is. Since the wind, especially strong wind, effectively disperses clouds, it notably increases SSD for the region as well).
- The correlation coeffienct between maxWIN and meanRHU was −0.639** and the quantitative influence of maxWIN on meanRHU (*ρ* value) was 0.576. So the wind factor has a strong negative influence on RHU.
- The correlation coeffienct between maxWIN and smallEVP was 0.633** and the quantitative influence of maxWIN on smallEVP (*ρ* value) was 0.602. So the wind factor has a strong positive influence on EVP.

The changing wind factor leads to the change of HUM, SSD and EVP conditions, which further influence local PM_2.5_ concentrations accordingly. As shown in Table 2, the correlation coefficient between SSD and PM_2.5_ concentration in winter was −0.715**, and the quantitative influence of SSD on PM_2.5_ concentration was 0.577 (*ρ* value), indicating the strong negative influence of SSD on PM_2.5_ concentration. By analogy, the correlation coefficient between meanRHU and PM_2.5_ concentration in winter was 0.759** and the quantitative influence of meanRHU on PM_2.5_ concentration was 0.738 (*ρ* value), indicating the strong positive influence of RHU on PM_2.5_ concentration. The correlation coefficient between smallEVP and PM_2.5_ concentration in winter was −0.494** and the quantitative influence of EVP on PM_2.5_ concentration was 0.287 (*ρ* value), indicating the comparatively strong negative influence of EVP on PM_2.5_ concentration.

According to the strong influences of wind factor on local PM_2.5_ concentration and strong interactions between wind factor and other meteorological factors, which also exert notable influences on PM_2.5_ concentration, the change of wind condition can be a promising meteorological mean for improving local air quality. By analogy, the change of SSD, RHU, EVP, Precipitation and other meteorological factors can also lead to significant change of local PM_2.5_ concentration.

In spite of the dominant role of energy conservation and emission reduction in improving local air quality, the significant influence of meteorological factors on PM_2.5_ concentration should be given enough emphasis. More research should be conducted to understand the complicated mechanism how different meteorological factors influence local PM_2.5_ concentration comprehensively. Meanwhile, researchers and decision makers should work together to design and employ feasible meteorological means, which may adjust local humidity, wind, precipitation or so forth, for improving local and regional air quality.

## Materials and Methods

### Data sources

The data of PM_2.5_ concentration are acquired from the website PM25.in. This website collects official PM_2.5_ data published by China National Environmental Monitoring Center (CNEMC) and provides hourly air quality information for all monitoring cities. Before Jan 1^st^, 2015, PM25.in publishes data of 190 monitoring cities. Since Jan 1^st^, 2015, the number of monitoring cities has increased to 367. By calling specific API provided by PM25.in, we have collected hourly PM_2.5_ data for these target cities since Jan 8^th^, 2014. The daily PM_2.5_ concentration for each city was calculated by averaging hourly PM_2.5_ concentration measured at all available local observation stations. The meteorological data for each city are obtained from the China Meteorological Data Sharing Service System (http://data.cma.cn/)s. The meteorological data provided by this website are compiled through thousands of observation stations across China. The meteorological observations include precipitation, temperature, wind speed, humidity and so forth. For this research, we obtained meteorological data for each city from Jan 1^st^, 2014 to Dec 31^st^, 2014. Based on the available PM_2.5_ and meteorological data, the study period for this research was set from Jan 8^th^, 2014 to Dec 31^st^, 2014.

## Methods

This research mainly aims to quantify the causality influence of individual meteorological factors on local PM_2.5_ concentration in the Jing-Jin-Ji region. Firstly, Pearson correlations between a set of meteorological parameters and local PM_2.5_ concentration are examined. As introduced, interactions between different meteorological factors are complicated and it can be highly difficult to quantify the influence of individual meteorological factors on PM_2.5_ concentration through correlation analysis. Therefore, correlation analysis works to preliminarily filter some meteorological factors that are not correlated with PM_2.5_ concentration and provide information for the following comparison. Meteorological factors correlated with PM_2.5_ concentration do not necessarily influence local air quality. Instead, some correlations may result from the underlying relationship between these factors and one agent factor^37^. To quantify the causality influence of individual meteorological on PM_2.5_ concentration and examine the performance of correlation analysis in complicated atmospheric environment, a robust approach for quantitative causality analysis is required.

Sugihara *et al*.^37^ suggested that mirage correlations might not be detected using correlation analysis. To detect the causality in complex ecosystems, Sugihara *et al*.^37^ proposed a convergent cross mapping (CCM) method. Different from Granger causality (GC) analysis^38^ that can be problematic in systems with weak to moderate coupling, the CCM algorithm is suitable for identifying causation in ecological time series. To examine the reliability of the CCM method under different situations, Sugihara *et al*.^37^ conducted a series of simple model experiments and field experiments, proving that the CCM approach effectively detects mirage correlation and reveals underling causality.

Since there are underlying interactions between individual meteorological factors, individual meteorological factors influence local PM_2.5_ concentration through complicated mechanisms. Furthermore, compared with Granger causality and forward-only dynamic time-warping (DTW), CCM method considers feedback relationship and thus reveals bidirectional causality^39^. Since heavily concentrated PM_2.5_ may also have a feedback effect on local meteorology, the CCM method is highly suitable for detecting potential bidirectional interactions between PM_2.5_ concentration and meteorological factors.

In this research, only several parameters need to be set for running this algorithm: E (number of dimensions for the attractor reconstruction), *τ* (time lag) and b (number of nearest neighbors to use for prediction). The value of E can be 2 or 3. A larger value of E produces more accurate convergent maps. The variable b is determined by E (b = E + 1). A small value of *τ* leads to a fine-resolution convergent map, yet requires much more processing time. Through a diversity of experiments, it was noted that the adjustment of these parameters simply affected some details of convergent maps whilst the general shape and information of curves remained unchanged. This indicates that the CCM method is not sensitive to manual setting of parameters and can extract reliable causality between different variables. In this research, to acquire optimal presentation effects of convergent cross maps, the value of *τ* was set as 2 days and the value of E was set 3.

## Additional Information

**How to cite this article**: Chen, Z. *et al*. Detecting the causality influence of individual meteorological factors on local PM_2.5_ concentration in the Jing-Jin-Ji region. *Sci. Rep.* **7**, 40735; doi: 10.1038/srep40735 (2017).

**Publisher's note:** Springer Nature remains neutral with regard to jurisdictional claims in published maps and institutional affiliations.

## Figures and Tables

**Figure 1:**
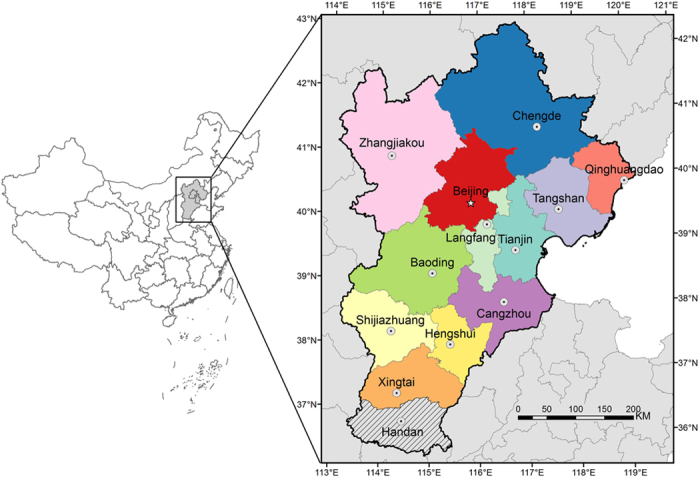
Geographical locations of cities in the Jing-Jin-Ji region. Handan is not included into our analysis due to lack of consistent meteorological data. The maps were drawn by the software of ArcGIS version 10.2, http://www.esri.com/software/arcgis/arcgis-for-desktop.

**Table 1:** Seasonal and overall mean daily PM_2.5_ concentration for different cites in the Jing-Jin-Ji region (μg/m^3^).

	Spring		Summer		Autumn		Winter		Overall	
	Mean	SD	Mean	SD	Mean	SD	Mean	SD	Mean	SD
Beijing	82.95	55.69	69.70	47.33	83.92	73.05	100.15	87.14	85.23	69.83
Tianjin	88.14	41.38	64.08	25.60	77.03	50.75	111.19	75.88	86.97	56.84
Shijiazhuang	101.37	51.71	83.70	44.64	98.62	79.31	180.72	123.01	121.52	94.10
Baoding	89.47	46.34	73.84	35.72	113.39	80.96	194.94	122.30	128.81	99.61
Tangshan	97.56	51.63	77.78	36.04	83.68	55.79	129.56	86.09	99.72	65.81
Qinghuangdao	55.14	34.39	39.17	21.31	53.16	41.68	80.92	56.35	59.94	45.43
Chengde	44.92	29.24	44.15	30.51	52.82	45.17	67.36	55.38	53.52	43.73
Zhangjiakou	26.83	14.03	20.81	10.73	22.27	13.33	58.33	63.38	34.36	40.66
Xingtai	109.70	50.32	78.60	37.61	115.82	91.13	193.11	117.19	129.55	95.14
Hengshui	85.89	41.31	73.03	27.96	98.03	53.74	147.81	86.94	107.86	68.84
Langfang	86.45	50.76	65.81	35.18	92.35	72.45	114.15	111.42	90.40	81.51
Cangzhou	80.00	38.62	58.16	24.88	76.29	48.61	117.87	69.41	88.28	56.51

**Table 2:** Seasonal correlations and causality between individual meteorological factors and PM_2.5_ concentration for different cities.

City	Spring	Summer	Autumn	Winter
Beijing	meanRHU** (0.532, 0.490)	minRHU** (0.648, 0.546), SSD** (−0.447, 0.324), minTEM** (0.554, 0.455),	meanRHU** (0.587, 0.555), SSD** (−0.509, 0.410), maxWIN** (−0.468, 0.223),	smallEVP** (−0.494, 0.287), meanRHU** (0.738, 0.738), SSD** (−0.715, 0.577), maxWIN** (−0.558, 0.531)
Tianjin	smallEVP** (−0.494, 0.428), meanRHU** (0.448, 0.226), extWIN** (−0.498, 0.349)	minTEM* (0.383, 0.118)	meanRHU** (0.442, 0.370)	smallEVP** (−0.478, 0.371), meanRHU** (0.554, 0.599), SSD** (−0.559, 0.493), maxWIN** (−0.485, 0.520)
Shijiazhuang	meanRHU** (0.575, 0.502), meanWIN* (−0.398, 0.322)	minRHU** (0.448, 0.359), SSD** (−0.516, 0.387)	meanRHU* (0.428, 0.225), extWIN** (−0.476, 0.293), SSD** (−0.477, 0.304)	smallEVP** (−0.414, 0.347), meanRHU** (0.494, 0.509), SSD** (−0.494, 0.565)
Baoding	smallEVP** (−0.454, 0.404), meanRHU** (0.496, 0.437)	minTEM** (0.523, 0.291)	minRHU* (0.415, 0.166), SSD* (−0.429, 0.221),	smallEVP** (−0.519, 0.299), meanRHU** (0.592, 0.597), SSD** (−0.592, 0.511), extWIN** (−0.498, 0.432)
Tangshan	smallEVP** (−0.473, 0.436), meanRHU** (0.500, 0.330), maxWIN* (−0.410, 0.46)	minTEM** (0.425, 0.257)	meanRHU* (0.408, 0.509)	smallEVP** (−0.435, 0.297), extWIN** (−0.562, 0.488)
Qinghuangdao	smallEVP** (−0.510, 0.440)	meanTEM* (0.365, 0.132)	SSD** (−0.441, 0.312)	smallEVP** (−0.431, 0.330), meanRHU** (0.593, 0.560), SSD** (−0.575, 0.423), maxTEM** (0.410, 0.217), extWIN** (−0.402, 0.362)
Chengde		minRHU** (0.480, 0.317), minTEM** (0.686, 0.640)	SSD** (−0.447, 0.216)	smallEVP** (−0.407, 0.214), meanRHU** (0.696, 0.530), SSD** (−0.596, 0.51), extWIN** (−0.422, 0.369), dir_maxWIN** (−0.379, 0.333), minTEM** (−0.412, 0.244)
Zhangjiakou	SSD** (−0.488, 0.325)	meanRHU** (0.510, 0.354), SSD** (−0.334, 0.08), minTEM** (0.424, 0.386)	minRHU* (0.431, 0.350)	SSD** (−0.468, 0.497), meanRHU** (0.565, 0.455), minTEM* (0.352, 0.306), extWIN** (−0.423, 0.508), dir_maxWIN* (−0.362, 0.441)
Xingtai	meanRHU** (0.483, 0.377)	maxPRS* (−0.372, 0.282), dir_extWIN** (0.401, 0.166)	SSD* (−0.409, 0.302)	meanRHU** (0.554, 0.455), SSD** (−0.553, 0.410)
Hengshui	smallEVP** (−0.478, 0.550), meanRHU** (0.514, 0.580), meanWIN** (−0.494, 0.480)	meanRHU* (0.444, 0)	meanRHU* (0.470, 0.234)	smallEVP** (−0.437, 0.237), minRHU** (0.518, 0.333), SSD** (−0.697, 0.343), extWIN** (−0.560, 0.288)
Langfang	meanRHU* (0.409, 0.387), extWIN* (−0.407, 0.558)	minTEM** (0.484, 0.289)	meanRHU** (0.470, 0.394), SSD** (−0.458, 0.273), extWIN** (−0.498, 0.361)	smallEVP** (−0.515, 0.301), meanRHU** (0.659, 0.606), SSD** (−0.697, 0.593), extWIN** (−0.560, 0.527)
Cangzhou	meanRHU** (0.579, 0.414), meanWIN** (−0.467, 0.457)	SSD (NA, 0.246)	meanRHU (NA, 0.081)	meanRHU** (0.492, 0.432), minRHU** (0.535, 0.414), SSD** (−0.582, 0.51)

**Correlation is significant at the 0.01 level (2 tailed); *Correlation is significant at the 0.05 level (2 tailed).

The first value in the brackets presents the correlation coefficient between the meteorological factor and PM_2.5_ concentration.

The second value presents the quantitative influence of individual meteorological factors on local PM_2.5_ concentration (*ρ* value), whilst the feedback effects of PM_2.5_ on these meteorological factors are not listed here.

For each cell in Table 2, only strongly correlated factors are listed. If there are several strongly correlated variables (e.g. meanWIN and maxWIN), which belong to the same meteorological category, then only the one with the largest correlation coefficient is listed.

NA indicates that no significant correlation exists between the meteorological factor and PM_2.5_ concentration.
